# Molecular Networking Reveals Antioxidant Properties and Phenolic Profiles of Four Rosaceae Seeds

**DOI:** 10.3390/plants14243749

**Published:** 2025-12-09

**Authors:** Mi Jeong Lim, Jinyoung Park, Min Sung Lee, Seong Yeon Choi, Heejung Yang, Taewan Kim, Chae Sun Na

**Affiliations:** 1 Forest Bioresources Department, Baekdudaegan National Arboretum, Bonghwa 36209, Republic of Korea; alwjd3191@koagi.or.kr (M.J.L.); next9101@koagi.or.kr (M.S.L.); 2 Laboratory of Natural Products Chemistry, College of Pharmacy, Kangwon National University, Chuncheon 24341, Republic of Korea; jypark@kangwon.ac.kr (J.P.); sych426@kangwon.ac.kr (S.Y.C.); heejyang@kangwon.ac.kr (H.Y.); 3 Department of Food Science and Biotechnology, Andong National University, Andong 36729, Republic of Korea; tk37@andong.ac.kr

**Keywords:** *Duchesnea indica*, antioxidant activity, GNPS, shikimates, phenylpropanoids, ellagic acid

## Abstract

For centuries, four Rosaceae species—*Malus sieboldii*, *Sorbus commixta*, *Duchesnea indica*, and *Prunus sargentii*—have been prized for their pharmacological properties. In this study, solvent extracts from the seeds of these species were prepared, and their total polyphenol and flavonoid contents were measured. Antioxidant capacity was evaluated using 1,1-diphenyl-2-picrylhydrazyl (DPPH) and 2,2′-azino-bis(3-ethylbenzothiazoline-6-sulfonic acid) (ABTS) radical scavenging assays, as well as ferric reducing antioxidant power (FRAP) and Fe^2+^ chelation tests. Compounds in the extracts were identified through molecular networking with the Global Natural Products Social Molecular Networking (GNPS) platform. Among all samples, *D. indica* extract contained the highest polyphenol and flavonoid concentrations (335.63 ± 0.03 mg gallic acid equivalents (GAE) per gram of extract and 230.14 ± 2.90 mg rutin equivalents (RE) per gram of extract, respectively). It also exhibited the strongest antioxidant activity in DPPH, ABTS, and FRAP assays, with statistically significant outcomes. Liquid chromatography/mass spectrometry analysis and molecular networking revealed a diverse metabolite profile corresponding to seven biosynthetic pathways in the extracts. Notably, *D. indica* extract was rich in shikimates, phenylpropanoids, and ellagic acid derivatives, which have potent antioxidant effects. These results suggest a strong relationship between the extract’s chemical profile and its biological activity, offering promising opportunities to use *D. indica* seeds as functional ingredients across various fields.

## 1. Introduction

Reactive oxygen species (ROS) are generated by normal cellular metabolism or exposure to various exogenous factors [1]. However, when ROS production overwhelms the antioxidant defense systems, oxidative stress occurs [2]. This imbalance impairs cellular functions and triggers apoptosis, primarily by inducing lipid peroxidation in cell membranes, leading to protein denaturation and DNA damage [3]. Chronic oxidative stress not only accelerates the aging process but is also recognized as a primary contributor to degenerative diseases, such as Alzheimer’s disease, cardiovascular disorders, diabetes, and other serious health conditions [4]. Hence, antioxidants have garnered increased attention as effective agents for managing oxidative stress and protecting biological systems from the detrimental effects of excessive ROS [5].

Synthetic antioxidants—such as butylated hydroxytoluene (BHT), butylated hydroxyanisole (BHA), and *tert*-butylhydroquinone (TBHQ)—have been widely employed in various applications [6]. However, since the 1990s, concerns have arisen regarding their safety, with reports documenting adverse effects on human health, including hepatotoxicity, endocrine disruption, and neurotoxicity [7,8]. This has fueled global interest in naturally derived bioactive materials as safer alternatives [9,10], with diverse biological activities. Notably, polyphenols and flavonoids from natural sources are effective antioxidants that help prevent cell damage by scavenging ROS [11]. Current research is focusing on natural antioxidants with anti-inflammatory, anticancer, and liver-protective properties. As a result, these natural products are attracting attention for their potential use in the growing dietary and pharmaceutical industries [12,13,14].

The Rosaceae family represents a large and globally significant group of angiosperms, comprising approximately 3200 species across numerous genera [15,16]. In Korea, this family is represented by four subfamilies, 35 genera, and 207 species, including economically and culturally important genera such as *Fragaria* (strawberry), *Malus* (apple), and *Rosa* (rose) [17,18]. Many Rosaceae plants have historically been utilized as edible and medicinal resources in traditional medicine practices [19,20]. For example, the leaves and branches of *Malus sieboldii* (Regel) Rehder exhibit antioxidant and antibacterial properties [21,22]. Similarly, the fruits, stems, and other tissues of *Sorbus commixta* Hedlund, *Duchesnea indica* (Andrews) Focke, and *Prunus sargentii* Rehder are recognized in traditional and modern research for their antioxidant, anti-inflammatory, and protective effects [23,24,25,26]. Despite ongoing research into the biological activities of whole-plant and fruit extracts from these Rosaceae species, there remains a lack of studies specifically examining the functional substances in their seeds. Based on findings from other plant parts, it is hypothesized that the seeds also harbor natural pharmacological components with significant potential.

To comprehensively investigate the complex composition of these seeds, metabolomics has become essential for yielding biochemical insights into metabolic profiles and distinguishing differences between species [27]. Recently, molecular networking (MN)—particularly via the Global Natural Product Social (GNPS) platform—has been recognized as a highly efficient tool for rapid metabolite identification using non-targeted mass spectrometry (MS) or MS-based data during early stages of natural product research [28]. The GNPS platform, an open-source web-based tool, facilitates raw data analysis (including data composition, processing, and fragmentation annotation) and supports the storage and sharing of processed data, enabling the construction of extensive chemical networks [29]. This approach facilitates comprehensive analyses of metabolite composition differences among various natural products and can be employed in chemical profiling to elucidate the unique characteristics and pharmacological properties of materials [30].

Therefore, the present study was designed to assess the antioxidant activity of seed extracts from four indigenous Rosaceae species (*M. sieboldii*, *S. commixta*, *D. indica*, *P. sargentii*) and to systematically compare metabolite differences among the varieties. By elucidating the chemical profiles of the extracts using MN technology, this research aims to provide foundational data that demonstrate their potential as naturally derived functional materials.

## 2. Results and Discussion

### 2.1. Yield, Total Polyphenol, and Total Flavonoid Content

The yields and total polyphenol and flavonoid contents of the four Rosaceae seed extracts were evaluated (Table 1). The extraction yields ranged from 3.04 ± 0.00 to 7.77 ± 0.01%, with *S. commixta* showing the highest yield at 7.77 ± 0.01%.

The total polyphenol content (TPC) of these extracts ranged between 74.95 ± 0.02 and 335.63 ± 0.03 mg gallic acid equivalents (GAE) per gram of extract. Specifically, the *D. indica* extract contained the highest TPC (335.63 ± 0.03 mg GAE/g), approximately 3.2 to 4.5 times that of the other species. The observed TPC values were consistent with previous studies; for example, Xu et al. reported high phenolic content in the methanol extract of whole *D. indica* [31], and Kim et al. measured a similar value in the fruit of *S. commixta* (335.63 ± 0.03 mg GAE/g) [32]. Polyphenol content in *M. sieboldii* leaves and branches was also reported to be 81.3 ± 1.2 mg GAE/g in an ethanol extract [33].

When considering total flavonoid content, the *D. indica* extract exhibited the highest value at 230.14 ± 2.90 mg rutin equivalents (RE) per gram of extract. The following order was observed: *S. commixta* (69.25 ± 1.75 mg RE/g), *M. sieboldii* (24.73 ± 0.41 mg RE/g), and *P. sargentii* (10.86 ± 0.22 mg RE/g). This distribution resembled the pattern observed for total polyphenol content. The proportion of flavonoids relative to total polyphenols showed considerable variation, from 14.14% in *P. sargentii* to 68.58% in *D. indica*. Such differences are attributed to environmental factors and genetic diversity among species [34].

Furthermore, significant differences in the accumulation and chemical composition of secondary metabolites among these species have been reported as contributing factors to the observed variability. Accordingly, these quantitative differences in phenolic content are expected to impact the overall antioxidant capacity of the extracts [35].

### 2.2. DPPH, ABTS Radical Scavenging Activity, and FRAP Assay

The antioxidant activities of the Rosaceae seed extracts were evaluated using 1,1-diphenyl-2-picrylhydrazyl (DPPH) and 2,2′-azino-bis(3-ethylbenzothiazoline-6-sulfonic acid) (ABTS) radical scavenging assays, alongside the ferric reducing antioxidant power (FRAP) assay (Table 2). The DPPH radical scavenging activity was expressed as half-maximal inhibitory concentration (IC_50_) values and compared with the positive control, L-ascorbic acid (19.27 ± 0.51 μg/mL). *D. indica* demonstrated the highest radical scavenging activity (106.50 ± 1.42 μg/mL), followed by *S. commixta* (423.79 ± 13.93 μg/mL), *M. sieboldii* (626.58 ± 19.73 μg/mL), and *P. sargentii* (927.90 ± 29.13 μg/mL). Flavonoids, which were abundant in *D. indica* (Table 2), exhibit robust antioxidant activity in DPPH assays [36]. Previous studies have also reported strong radical-scavenging and H_2_O_2_ neutralization activities in *D. indica* fruit [37], supporting the results of this study.

Results from the ABTS assay confirmed IC_50_ values ranging from 10.24 ± 0.02 to 219.65 ± 1.20 μg/mL. Again, *D. indica* exhibited the highest antioxidant activity (10.24 ± 0.02 μg/mL), while *P. sargentii* had the lowest (219.65 ± 1.20 μg/mL). The DPPH and ABTS scavenging activities were strongly correlated, with a similar trend observed across the extracts: *D. indica* and *S. commixta* showed high radical-scavenging potential. Increased scavenging activity was associated with higher phenolic compound content, reflecting the recognized link between phenolic content and antioxidant capacity [38].

For the FRAP assay, measurements at 50 μg/mL of seed extract ranged between 301.11 ± 3.75 and 3950.47 ± 21.48 μM. *D. indica* exhibited the highest FRAP activity (3950.47 ± 21.48 μM), followed by *S. commixta* (619.01 ± 0.62 μM). No significant differences were observed between *P. sargentii* (356.07 ± 5.44 μM) and *M. sieboldii* (301.11 ± 3.75 μM). These results support previous findings indicating a strong correlation between FRAP activity and radical-scavenging activity through electron donation [39]. The higher FRAP value of *D. indica* aligns with its superior radical-scavenging capacity. Overall, antioxidant activity increased alongside total polyphenol content, consistent with existing literature [40].

The antioxidant assays confirmed that *D. indica* seed extract possessed the strongest antioxidant activity among the Rosaceae species evaluated. Significant interspecies differences were observed, attributable to varying levels of antioxidant compounds. These findings prompted further investigation into the chemical profiles of the seed extracts to elucidate the basis for the observed differences in antioxidant activity.

### 2.3. LC–MS Data Analysis

**Figure 1:**
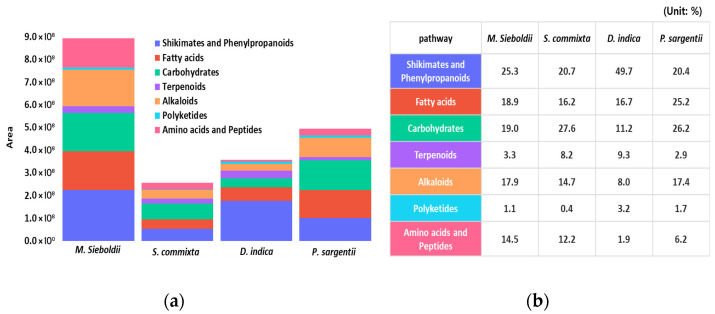
Quantitative and relative compositional analysis of chemical abundance in four Rosaceae species’ seed extracts by negative ion mode LC–MS and SIRIUS classification. (**a**) Quantitative comparative analysis showing the absolute peak area of major metabolite classes; (**b**) Relative compositional ratios (Unit: %) of the major metabolite classes across the four species.

Ultra-performance liquid chromatography-tandem mass spectrometry (UPLC–MS/MS) in negative ion (NI) mode was used to detect Rosaceae seed metabolites, with total ion chromatograms presented in Figure 1. In this mode, 1600 features were detected across the four Rosaceae seed species and categorized into seven chemical groups: shikimates and phenylpropanoids, fatty acids, carbohydrates, terpenoids, alkaloids, polyketides, and amino acids/peptides.

Upon examination of the compositional ratios based on relative peak areas, shikimates and phenylpropanoids were most prevalent in *D. indica*. Fatty acids dominated in *P. sargentii*, while carbohydrates were abundant in *S. commixta* and *P. sargentii*. Terpenoids were notably present in *S. commixta* and *D. indica*; alkaloids were more abundant in *M. sieboldii* and *P. sargentii*. Amino acids and peptides had relatively high ratios in *M. sieboldii* and *S. commixta* (Figure 1).

**Figure 2:**
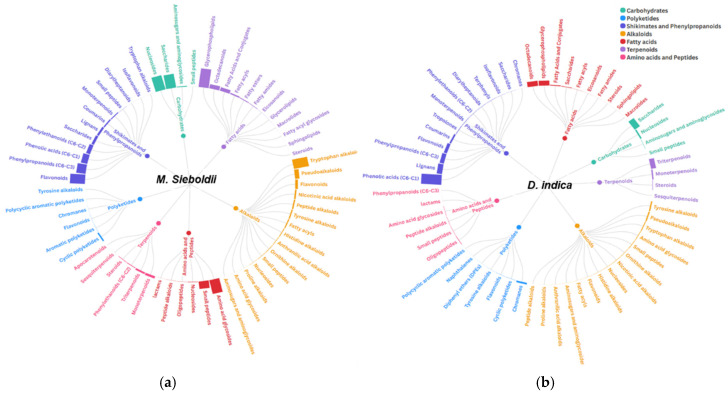
Comparative LC–MS/MS-based sunburst chart analysis of differential metabolite composition in *Malus sieboldii* and *Duchesnea indica* seed extracts, constructed using the Flourish platform. The hierarchical organization of metabolite classes is visualized using a radial tree diagram format. Relative abundances (fractional proportion) of specialized metabolite classes in the seed extracts of (**a**) *M. Sieboldii* and (**b**) *D. indica*. The size and color of each segment directly correspond to the relative contribution of each metabolite class to the total detected compounds.

Based on the compositional analysis, the physiological activities of the seed extracts were assessed. *D. indica* exhibited the highest total phenolic and antioxidant activity, whereas *M. sieboldii* showed a richer overall chemical composition according to LC–MS metabolome profiling. To elucidate the differential effects of composition on physiological activity, the metabolite profiles of the high-compositional-richness *M. sieboldii* and the highly antioxidant-active *D. indica* were compared in detail (Figure 2).

*Malus sieboldii* exhibited a broad metabolic composition, including shikimates/phenylpropanoids (28 features, 25.3% of total peak area), fatty acids (22 features, 18.9%), and carbohydrates (12 features, 19.0%), which suggests high chemical diversity. However, primary metabolites such as amino acids, fatty acids, and carbohydrates primarily function as energy sources for plant cells and do not contribute directly to antioxidant activity [41]. Consequently, despite possessing a wide-ranging metabolite profile, the antioxidant capacity of *M. sieboldii* was observed to be relatively lower.

The *D. indica* extract was characterized by 32 shikimate/phenylpropanoid metabolite features, with their total peak area ratio accounting for 49.7%. Shikimate/phenylpropanoid pathway derivatives are secondary metabolites featuring multiple hydroxyl groups (–OH), which are known for their powerful antioxidant properties, conferring strong ROS scavenging, protection against oxidative damage, and immunomodulatory activity [42,43]. Through the high concentration and accumulation of these antioxidant-active compounds, *D. indica* exhibited the highest antioxidant activity among the four species.

Collectively, these findings suggest that the difference in antioxidant activity between the seed extracts is determined by the concentration of antioxidant compounds like shikimates and phenylpropanoids, rather than by mere chemical compositional diversity.

LC–MS analysis of the *D. indica* seed extract was subsequently performed to further analyze the detailed metabolite profile of this specific class, confirming that the profile was predominantly composed of phenolic compounds (Figure 2).

Major classes identified included phenolic acids, lignans, flavonoids, and coumarins. The phenolic acid group featured a substantial presence of gallotannins and simple phenolic acids; the lignan group included neolignans and four furanoid lignans. Within the flavonoid group, subclasses such as flavonols, chalcones, flavan-3-ols, and phenylpropanoids were detected. These results confirm that *D. indica* seeds are particularly rich in phenolic acids, compounds recognized for their strong antioxidant potential.

Phenolic acids, including gallotannins, are widely recognized for their potent antioxidant, antibacterial, antiproliferative, cardiovascular protective, and antidiabetic effects [44]. Flavonoids offer anti-allergic, antiviral, anti-inflammatory, anticancer, and vasodilatory effects, as well as notable free-radical scavenging properties. Coumarins, secondary metabolites produced in defense against pathogens, exhibit antimicrobial, anticoagulant, antihypertensive, and antifungal activities [45]. The major bioactive phenolic compounds identified here are consistent with prior reports on *D. indica*, as confirmed by nuclear magnetic resonance (NMR) spectroscopy [46,47,48].

**Figure 3:**
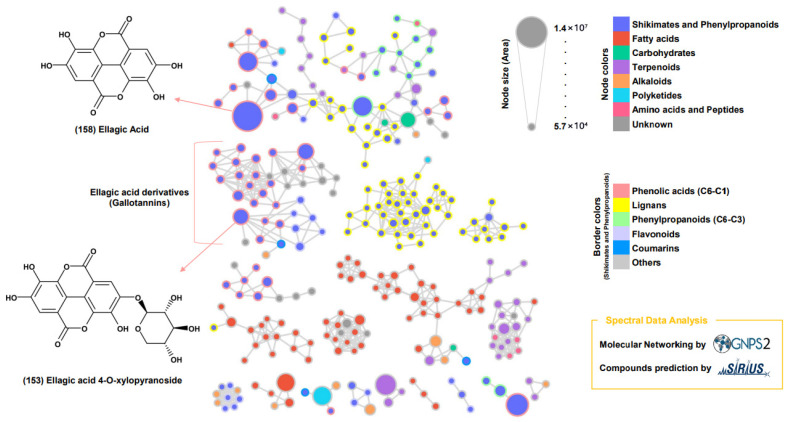
Molecular networking of metabolites from *Duchesnea indica* Extracts. Subnetworks from the molecular network of *D. indica* seed metabolites were created using GNPS2 spectral libraries, with compound prediction performed by SIRIUS. Node size corresponds to relative abundance, node colors represent major chemical classes, and border colors indicate subclasses.

**Figure 4:**
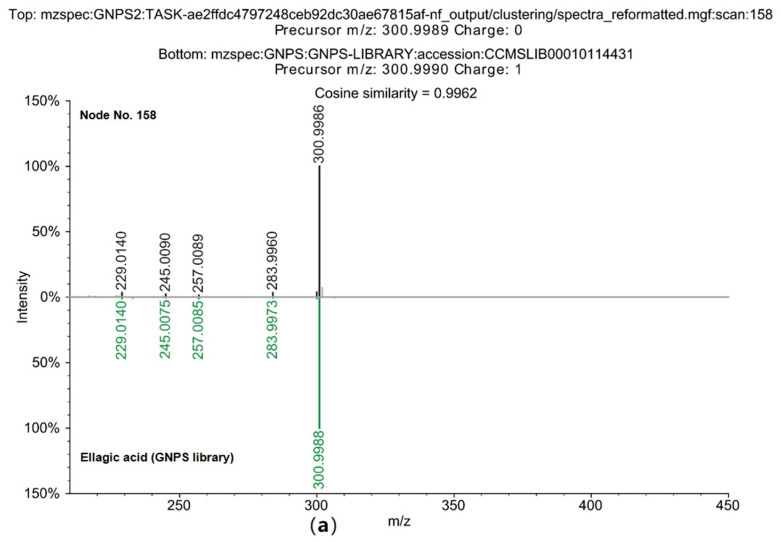

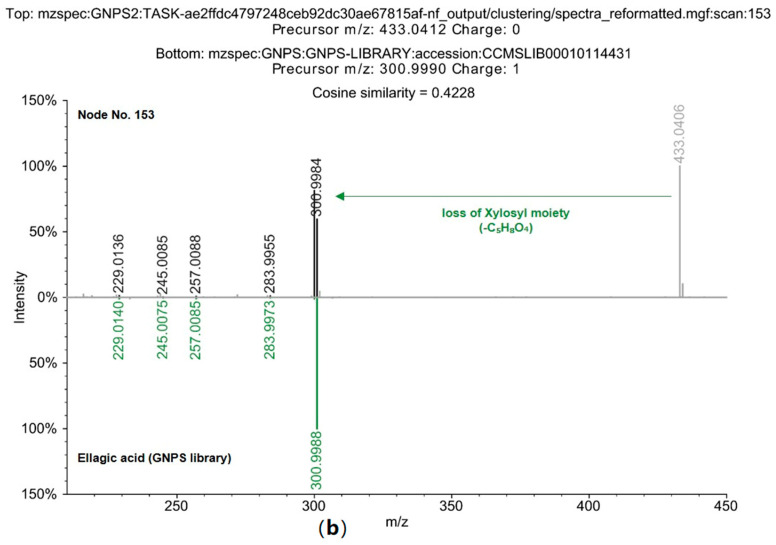
Confirmation of Ellagic acid identity via Mirror Plot Analysis of MS spectrum. (**a**) Comparative analysis of Node 158, tentatively identified as ellagic acid, with the GNPS2 library reference MS spectrum of authentic elllagic acid. (**b**) Comparative analysis of Node 153, tentatively identified as ellagic acid, with theGNPS2 library reference MS spectrum of authentic ellagic acids.

The GNPS spectrum library was used to analyze the MS/MS spectra of the extract’s compounds through feature-based MN and the SIRIUS program (Figure 3 and Figure 4). Network annotation propagation (NAP) analysis identified ellagic acid (158), a shikimate and phenylpropanoid derivative, as the largest node (Figure 3). Compound 153, which contained a xylosyl group, was classified in a separate cluster from ellagic acids (158).

Comparisons of the MS/MS pattern with the GNPS library (Figure 4) confirmed that the node ellagic acid (158) matched the standard spectrum with a cosine score of 0.9962, identifying it as a key marker compound in the phenolic profile of *D. indica* [49]. This result corroborates previous findings for the ethanol fraction of whole *D. indica*, where ellagic acid and its glycosides were quantitatively prominent (30.52 mg/100 g). The current GNPS analysis similarly confirmed the presence of these phenolics in *D. indica* seeds [50].

Certain Rosaceae species are known for high tannin content—such as ellagic acid (ellagitannin) and gallic acid (gallotannin)—which contribute significantly to their antioxidant activity. The MN analysis suggested that the major metabolites in *D*. *indica* seed extract were responsible for its strong antioxidant capacity [51].

Ellagic acid 4-O-xylopyranoside reportedly possesses antibacterial and anti-biofilm activity [52]. Ellagic Acid, characterized by two lactone rings and four hydroxyl groups, is recognized for its ability to scavenge ROS, neutralize pro-oxidants, and enhance antioxidant enzyme expression and activity, such as superoxide dismutase. As a key antioxidant, ellagic acid exhibits anti-inflammatory, antimutagenic, hepatoprotective, and neuroprotective effects [53,54]. Recent studies have highlighted its role in cell proliferation, apoptosis, DNA damage, and angiogenesis, identifying it as a potential anticancer agent [55].

In summary, the ellagic acid detected in *D. indica* seeds contributed significantly to radical scavenging and metal ion reduction, resulting in superior antioxidant activity compared to that of the other species. Hence, *D. indica* seeds are suggested as a promising resource not only for medicinal use but also as a valuable natural antioxidant.

## 3. Materials and Methods

### 3.1. Chemicals

Methanol, dimethyl sulfoxide (DMSO), Folin–Denis reagent, sodium carbonate (Na_2_CO_3_), tannic acid, aluminum nitrate, potassium acetate, quercetin, DPPH, ABTS, potassium persulfate, 2,4,6-tris(2-pyridyl)-s-triazine (TPTZ), ferric chloride (FeCl_3_·6H_2_O), and ferrous sulfate (FeSO_4_·7H_2_O) were purchased from Sigma-Aldrich (St. Louis, MO, USA). Ethanol was obtained from Duksan (Ansan, Republic of Korea). The ABTS radical was generated using ABTS and potassium persulfate, and the FRAP reagent was prepared from acetate buffer, TPTZ, and FeCl_3_·6H_2_O. Liquid Chromatography–MS/MS analysis was performed with distilled water and acetonitrile containing 0.1% formic acid as the mobile phase.

### 3.2. Materials

Four Rosaceae species (*M. sieboldii*, *S. commixta*, *D. indica*, and *P. sargentii*) were purchased from Simpol (Dongducheon, Republic of Korea)and stored at room temperature before being ground with a grinder (FM-909W, Hanil Co., Ltd., Sejong, Republic of Korea) for use as extraction samples.

### 3.3. Extraction and Yield Measurement

For extraction, 10 g of dried and ground seeds were combined with 200 mL of 70% methanol and stirred at room temperature for 48 h. The resulting mixture was passed through Advantec filter paper (No. 2, Advantec, Tokyo, Japan), concentrated under reduced pressure, freeze-dried, and then dissolved in DMSO for subsequent use. The extraction yields were calculated as the percentage of the raw material weight relative to the dried extract weight.

### 3.4. Total Polyphenol Content

Total polyphenol content was determined using the Folin and Denis (1915) method, which relies on the reaction of phenolic compounds with phosphomolybdic acid to produce a blue color [56]. Specifically, 50 μL of extract (1 mg/mL) was combined with 650 μL of purified water and 50 μL of Folin–Denis reagent (Sigma-Aldrich). The mixture was allowed to react at room temperature for 3 min. Subsequently, 100 μL of 10% Na_2_CO_3_ was added, mixed, and incubated at 37 °C for 1 h. Absorbance was measured at 725 nm using a microplate reader (MARK™; BIO-RAD, Hercules, CA, USA). A standard curve was generated using gallic acid (Sigma-Aldrich), and the total polyphenol content was calculated accordingly.

### 3.5. Total Flavonoid Content

Total flavonoid content was measured using the Moreno method [57]. The extract was diluted to various concentrations, and 100 μL of the extract was mixed sequentially with 20 μL of 10% aluminum nitrate (Sigma-Aldrich), 20 μL of 1 M potassium acetate (Sigma-Aldrich), and 860 μL of ethanol (Duksan). This mixture was allowed to react at room temperature for 40 min. Subsequently, the suspension was centrifuged to settle any precipitates. Next, 200 μL aliquots were transferred to a 96-well plate, and absorbance was measured at 415 nm. The experiment was repeated three times, and the results were averaged. A standard calibration curve was prepared using rutin (Sigma-Aldrich) to determine the total flavonoid content in the extracts.

### 3.6. DPPH Radical Scavenging Activity

DPPH radical-scavenging activity was evaluated using a modified version of the method described by Blois (1958) [58]. For the assay, 40 μL of each sample was mixed with 760 μL of a DPPH solution (5 mg/100 mL ethanol), diluted with ethanol to an absorbance of 1.0 at 515 nm. The mixture was reacted at 37 °C for 30 min, and the absorbance was measured at 515 nm using a UV/Visible spectrophotometer (Perkin Elmer, Waltham, MA, USA). The percentage of DPPH radical inhibition was calculated using the formula: Inhibition (%) = [(A0 − A)/A0], where A0 is the absorbance of the DPPH control, and A denotes the absorbance of the sample with DPPH. The IC_50_ value, indicating the concentration required to reduce DPPH free radical activity by 50%, was determined. A lower IC_50_ value reflected greater antioxidant potency.

### 3.7. ABTS Radical Scavenging Activity

The ABTS radical scavenging activity was assessed using a modified version of a previously described protocol [59]. Briefly, 7.4 mM ABTS (Sigma-Aldrich) and 2.45 mM potassium persulfate (Sigma-Aldrich) were combined at their final concentrations and incubated for 24 h to form ABTS^+^. The resulting solution was diluted with phosphate-buffered saline (PBS; Sigma-Aldrich, pH 7.4) to an absorbance of 0.70–0.80 at 734 nm. To 190 μL of the ABTS solution, 10 μL of the extract was added, followed by a 10 min reaction. Absorbance was measured at 734 nm using a microplate reader (iMARK™; BIO-RAD, Hercules, CA, USA). The inhibition percentage was calculated as Inhibition (%) = [(A0 − A)/A0], where A0 is the absorbance of the ABTS control and A denotes the absorbance of the sample. The IC_50_ was calculated, with lower IC_50_ values indicating higher antioxidant capacity.

### 3.8. FRAP Assay

The FRAP assay was performed according to the method of Benzie and Strain [60]. The FRAP reagent was prepared by mixing 25 mL of 300 mM acetate buffer (pH 3.6), 2.5 mL of 10 mM TPTZ (Sigma-Aldrich) dissolved in 40 mM HCl, and 2.5 mL of 20 mM FeCl_3_·6H_2_O solution. For the assay, 25 μL of the sample was mixed with 175 μL of the FRAP reagent, which had been pre-incubated at 37 °C for at least 10 min. The reaction proceeded in the dark at 37 °C for 30 min. Absorbance was measured at 590 nm using a spectrophotometer (Ultraspec 2100pro, Biochrom Ltd., Cambridge, UK). The FRAP value was calculated using a standard curve prepared by quantifying FeSO_4_·7H_2_O (Sigma-Aldrich).

### 3.9. LC–MS/MS Analysis

Metabolite analysis was performed using a Thermo Scientific Vanquish Flex UHPLC system (Waltham, MA, USA). Metabolites were separated at 25 °C using an ACQUITY UPLC^®^ BEH C18 column (50 × 2.1, 1.7 µm; Waters, Milford, MA, USA). The mobile phases comprised distilled water with 0.1% formic acid (A) and acetonitrile with 0.1% formic acid (B). The flow rate was set at 0.3 mL/min, and the injection volume was 5 µL. The mobile phase gradient was: 0–0.5 min, 10% B; 0.5–14.5 min, 10–90% B; 14.5–17 min, 90% B; 17–19.5 min, 90–10% B. A Thermo Fisher Orbitrap Exploris 120 mass spectrometer, coupled with the liquid chromatograph, was used. Operating parameters included an ionization voltage of 2500 V in NI mode, with the ion transfer tube at 325 °C and the Vaporizer at 350 °C. Gas flows were set to Sheath Gas 50 Arb, Aux Gas 10 Arb, and Sweep Gas 1 Arb. MS/MS analysis involved fragmentation of parent ions using HCD collision energies of 15%, 30%, and 60%.

### 3.10. LC–MS/MS Data Analysis

Raw LC data files were converted to Mass Spectrometry Markup Language (mzML) format using MSConvert software (version 3.0.25054) to ensure compatibility and facilitate data retrieval. The Ultra-Performance Liquid Chromatography–Tandem Mass Spectrometry (UPLC–MS/MS) data were preprocessed with Mzmine (ver. 3.9.0) software [61]. The data preprocessing sequence involved several critical steps: mass detection, ADAP chromatogram building, local minimum feature resolution, and the subsequent application of a ^13^C isotope filter. A join aligner was applied to all adjusted peak lists, followed by peak finding and filtering of feature-list rows. Additionally, to facilitate data interpretation, a radial tree diagram illustrating the hierarchical organization of metabolite classes was constructed using the Flourish platform (Flourish Studio, London, UK). Feature-based Molecular Networking (MN) was performed using the Global Natural Products Social Molecular Networking (GNPS2) platform, and the results were visualized in Cytoscape (ver. 3.10.1) [62].

### 3.11. Statistical Analysis

Experimental results are expressed as the mean ± standard deviation based on three independent measurements. Significant differences between data were tested at the *p* < 0.05 level using one-way Analysis of Variance (ANOVA) and Tukey’s test.

## 4. Conclusions

Although the Rosaceae family has a long history of use, there is a lack of comprehensive comparative research specifically examining the seeds of four species (*M. sieboldii*, *S. commixta*, *D. indica*, and *P. sargentii*). This study was conducted to address this gap by evaluating the antioxidant potential and specialized metabolite profiles of these seeds. Among the seeds investigated, *S. commixta* produced the highest extraction yield. *D. indica* seeds distinguished themselves with the highest total polyphenol (335.63 ± 0.03 mg GAE/g) and total flavonoid (230.14 ± 2.90 mg RE/g) levels. This high phytochemical load was directly linked to the outstanding in vitro antioxidant capacity observed for the *D. indica* seed extract, as demonstrated by the results from the DPPH, ABTS, and FRAP assays. Subsequent LC–MS/MS metabolomics and MN revealed that *D. indica* has a distinct metabolic signature, primarily featuring compounds from the shikimate and phenylpropanoid biosynthetic pathways. Ellagic acid derivatives were especially prevalent. The strong antioxidant activity of these phenolic compounds supports the potential of *D. indica* seed extract as a valuable functional ingredient for food and nutraceutical applications.

## Figures and Tables

**Table 1:** Total polyphenol and total flavonoid content of four Rosaceae species seed extracts.

Scientific Name	Yield (%)	Total Polyphenols (mg GAE/g of Extracts)	Total Flavonoids (mg RE/g of Extracts)
*Malus sieboldii*	6.55 ± 0.02 ^b^	74.95 ± 0.02 ^c^	24.73 ± 0.41 ^c^
*Sorbus commixta*	7.77 ± 0.01 ^a^	103.54 ± 0.02 ^b^	69.25 ± 1.75 ^b^
*Duchesnea indica*	3.04 ± 0.00 ^d^	335.63 ± 0.03 ^a^	230.14 ± 2.90 ^a^
*Prunus sargentii*	4.86 ± 0.01 ^c^	76.77 ± 0.02 ^c^	10.86 ± 0.22 ^c^

GAE, gallic acid equivalent; RE, rutin equivalent. Values are expressed as mean ± standard deviation (*n* = 3). Different letters within a column differ significantly (*p* < 0.05).

**Table 2:** Antioxidant activities in the seed extracts of four *Rosaceae* species.

Scientific Name	IC_50_ [μg/mL]		FRAP * (μM FeSO_4_ eq/mg of Extract)
	DPPH Radical Scavenging Activity	ABTS Radical Scavenging Activity	
Ascorbic acid	19.27 ± 0.51 ^a^	4.88 ± 1.53 ^a^	6815.38 ± 39.49 ^a^
*Malus sieboldii*	626.58 ± 19.73 ^d^	166.53 ± 8.43 ^d^	301.11 ± 3.75 ^d^
*Sorbus commixta*	423.79 ± 13.93 ^c^	99.29 ± 0.41 ^c^	619.01 ± 0.62 ^c^
*Duchesnea indica*	106.50 ± 1.42 ^b^	10.24 ± 0.02 ^b^	3950.47 ± 21.48 ^b^
*Prunus sargentii*	927.90 ± 29.13 ^e^	219.65 ± 1.20 ^e^	356.07 ± 5.44 ^d^

IC_50_, Concentration required to inhibit 50% of the DPPH and ABTS radical-scavenging activity; * FRAP, ferric reducing antioxidant power based on the reduction of Fe(II)/mg of extract. Values are expressed as mean ± standard deviation (*n* = 3). Different letters within a column differ significantly (*p* < 0.05).

## Data Availability

The original contributions presented in this study are included in the article. Further inquiries can be directed to the corresponding author.
